# Supplementary material for: WNT-FRIZZLED-LRP5/6 Signaling Mediates Posterior Fate and Proliferation during Planarian Regeneration
Source: Genes (Basel). 2021 Jan 15;12(1):101. doi: 10.3390/genes12010101 (PMC7830089; doi:10.3390/genes12010101)
Supplement: Supplementary file 1 [file genes-12-00101-s001.zip › Pascual_Sureda_20201231_SUPP.pdf]

Supplementary information

WNT-FRIZZLED-LRP5/6 signaling mediates posterior fate and proliferation during planarian regeneration.

Eudald Pascual-Carreras [(1) (2)+], Miquel Sureda-Gomez [(1) (2)+], Ramon Barull [(1) (2)], Natàlia Jordà [(1) (2)], Maria Gelabert [(1) (2)], Pablo Coronel-Cordoba [(1) (2)], Emili Saló* & Teresa Adell*

[(1)] Department of Genetics, Microbiology and Statistics and Institute of Biomedicine, Universitat de Barcelona, Barcelona, Catalunya, Spain.

[(2)] Institut de Biomedicina de la Universitat de Barce

lona (IBUB), Universitat de Barcelona, Barcelona, Catalunya, Spain.

[+] Equal contribution

* Corresponding authors: esalo@ub.edu and tadellc@ub.edu

Figure S1

Figure S2

Figure S3

Figure S4

Figure S5

Figure S6

Table Supplementary 1

Table Supplementary 1

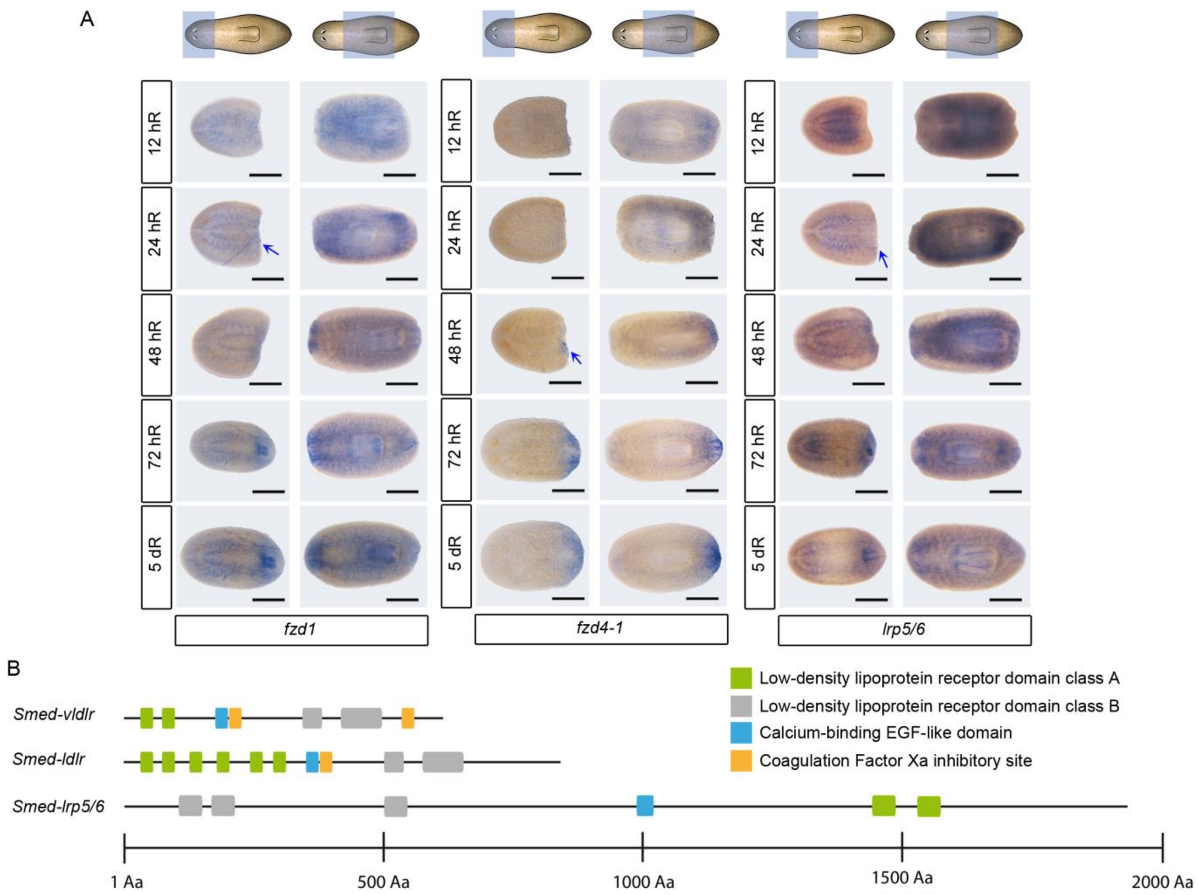

Figure S1. *fzd1*, *fzd4-1* and *lrp5/6* expression patterns in regenerating animals. (**A**) WISH of *fz1*, *fzd4-1* and *lrp5/6* at different hours of regeneration (hR) and days of regeneration (dR). Blue shadows in the schematic cartoons represent the regenerative pieces studied: heads, trunks and tails. Arrows point to the earliest detection of their expression in posterior wounds. (**B**) Schematic representation of the conserved domains found in *Smed-vldlr*, *Smed-ldlr* and *Smed-lrp5/6* proteins. Scale bar: 100 µm.

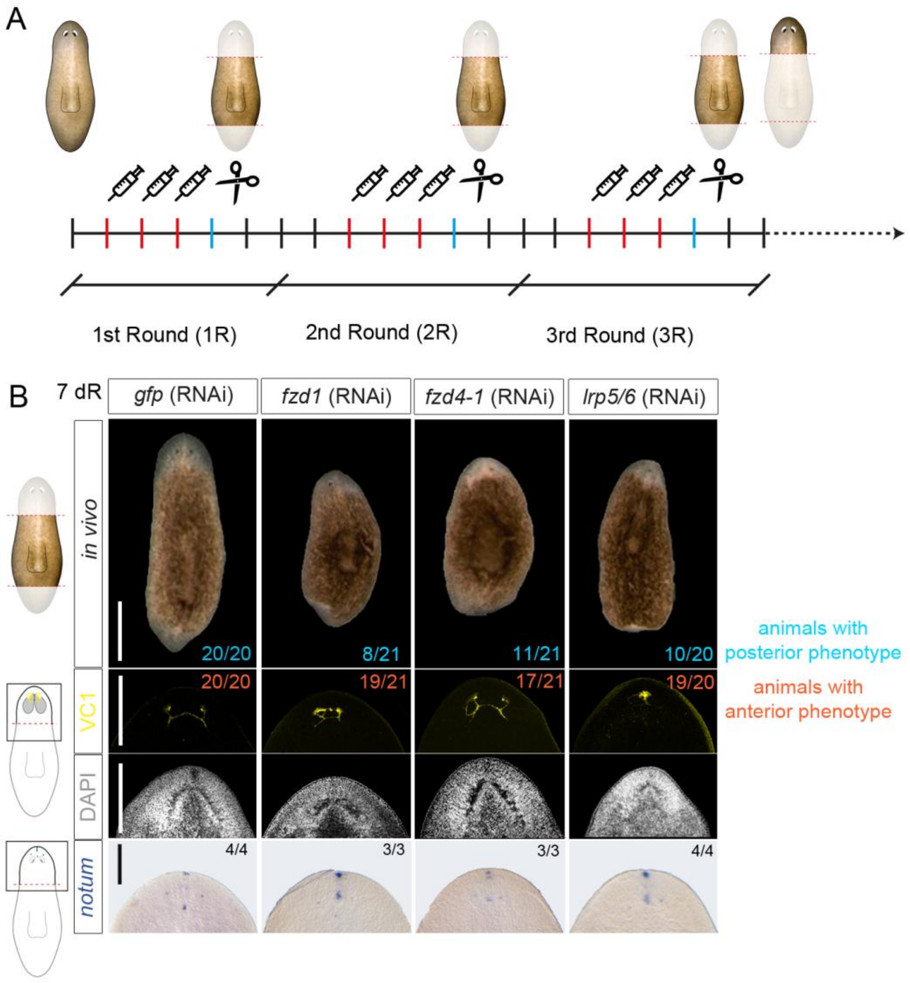

Figure S2. *fzd1*, *fzd4-1* and *lrp5/6* (RNAi) regenerating trunks present anterior and posterior defects. (**A**) Schematic illustration indicating the RNAi procedure of three rounds of inhibition and amputation. (**B**) *in vivo* images of planarians after the inhibition of the three receptors. They showed anterior and posterior defects. Anti-arrestin (VC-1) shows the defects in the visual system and nuclear staining (DAPI) shows the defects in the brain. WISH of *notum* demonstrates that the anterior pole is present. Scale bars: 150 µm.

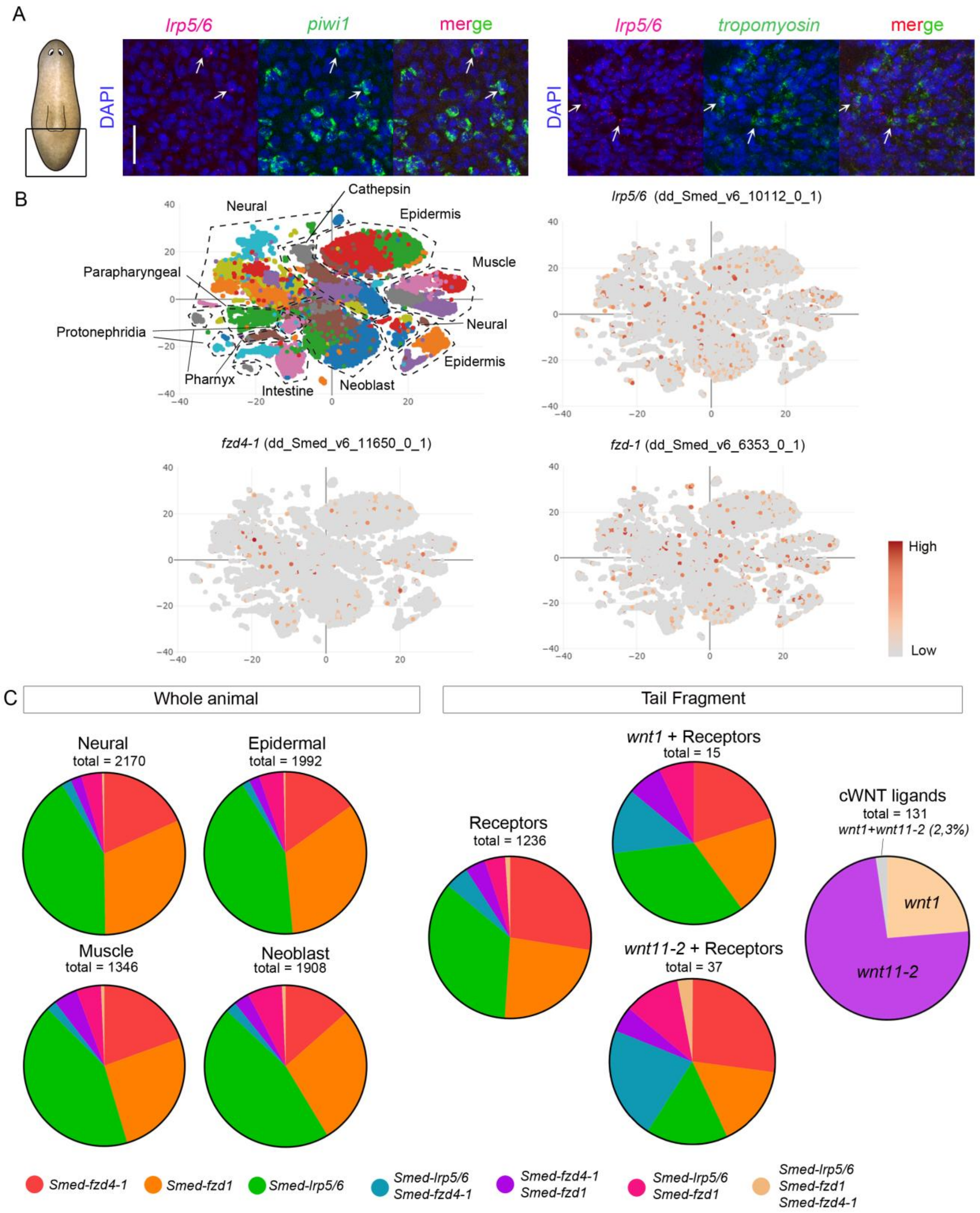

Figure S3. *fzd1* and *lrp5/6* are broadly expressed in intact animals and colocalize in different cell types. (**A**) Double FISH in intact animals showed the colocalization of *lrp5/6* with *piwi1* (neoblast marker) and *tropomyosin* (muscular marker). A schematic illustration indicates the region studied. Scale bar: 25 µm. (**B**) B) tSNE plots of fzd1, fzd4-1 and lrp5/6 showing their expression in all planarian cell types (**C**) Pie charts of Gene co-expression counts from the single cell sequencing database (27) using the platform https://compgen.bio.ub.edu/PlanNET/planexp. *fzd1*, *fzd4-1* and *lrp5/6* expression and their co-expression in neural, epidermal, muscle and neoblast cell types, was analyzed in the database corresponding to the whole animal (left). *fzd1*, *fzd4-1* and *lrp5/6* expression, the co-expression between them and with *wnt1* or *wnt11-2* was analyzed in the database corresponding to the tail fragments (right). The analysis also shows that *wnt1* and *wnt11-2* are coexpress in very few cells (2,3%).

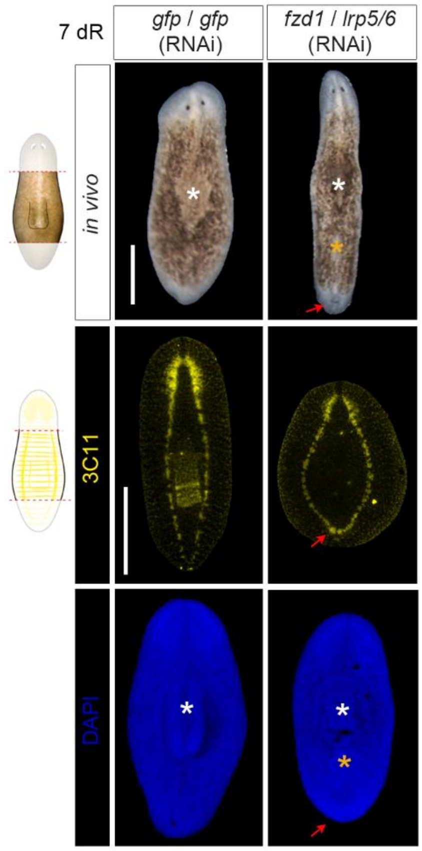

Figure S4. *fzd1 / lrp5/6* (RNAi) regenerating trunks showed a Two-head phenotype. Top: *in vivo* images of Two-head planarian after *fzd1 / lrp5/6* inhibition. Middle: Immunostaining using α-SYNAPSIN (3C11) (neural system) showed a second brain in the posterior part in *fzd1 / lrp5/6* (RNAi) trunks. Bottom: nuclei were stained with DAPI, showing two pharynges and a posterior brain. Red arrows indicate the second head. Asterisks mark the old (white) and the new (orange) pharynges. On the left side, schematic illustrations were added showing where amputation performed and (red dashed line) and the expression of 3C11 in intact animals. Scale bars: 500 µm.

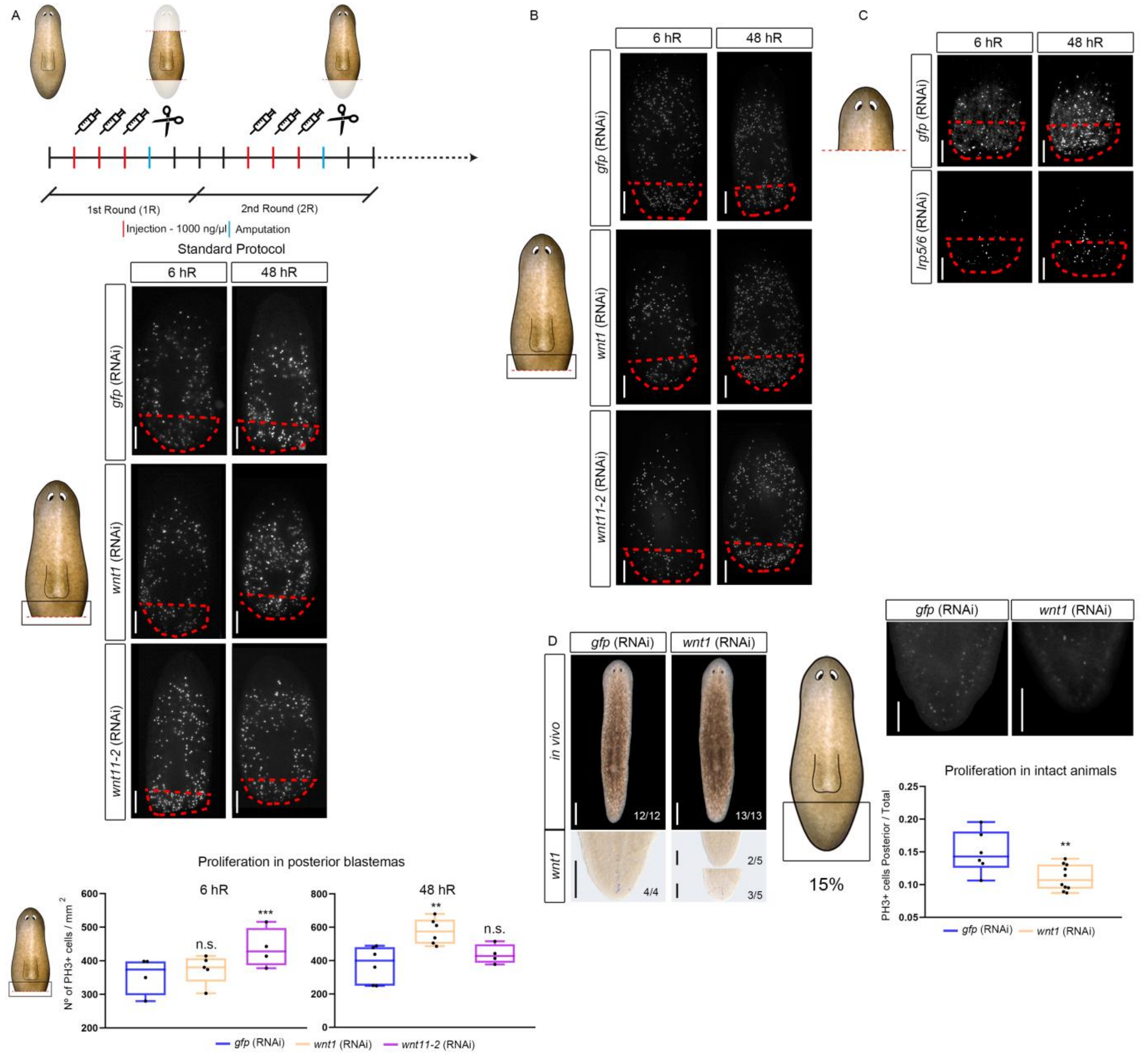

Figure S5. cWNT elements control proliferation in intact animals and during posterior regeneration. (**A**) Schematic illustration indicated the RNAi procedure by the usual protocol (two weeks of RNAi). The area quantified and the amputated area of the trunks are indicated with a dark box and dashed red line, respectively. Images of the immunostaining using α-PH3 antibody. Quantification of PH3+ cells after usual protocol (two weeks of RNAi) at 6 hours of regeneration (hR) (*gfp*, *n*= 4; *wnt1*, *n*= 5; *wnt11-2*, *n*= 6; ****P*<0.001, n.s.) and 48 hR (*gfp*, *n*= 6; *wnt1*, *n*= 6; *wnt11-2*, *n*= 5; ***P*<0.01, n.s.). (**B**) Images of the immunostaining using α-PH3 antibody of the regenerating trunks of *gfp*, *wnt1* and *wnt11-2* at 6 hR and 48 hR. (**C**) Images of immunostaining using α-PH3 antibody of regenerating trunks of *gfp* and *lrp5/6* at 6 hR and 48 hR. (**D**) *wnt1* (RNAi) animals do not show any *in vivo* phenotype but WISH of *wnt1* showed its absence or reduction in knockdown animals. Images of the immunostaining using α-PH3 antibody of the area quantified, indicated with a dark box. Quantification of PH3+ cells after one week of treatment showed a reduction in *wnt1* (RNAi) animals (*gfp*, *n*= 6; *wnt1*, *n*= 10; ***P*<0.01). Red dashed line indicates the area quantified. Scale bars: 100 µm.

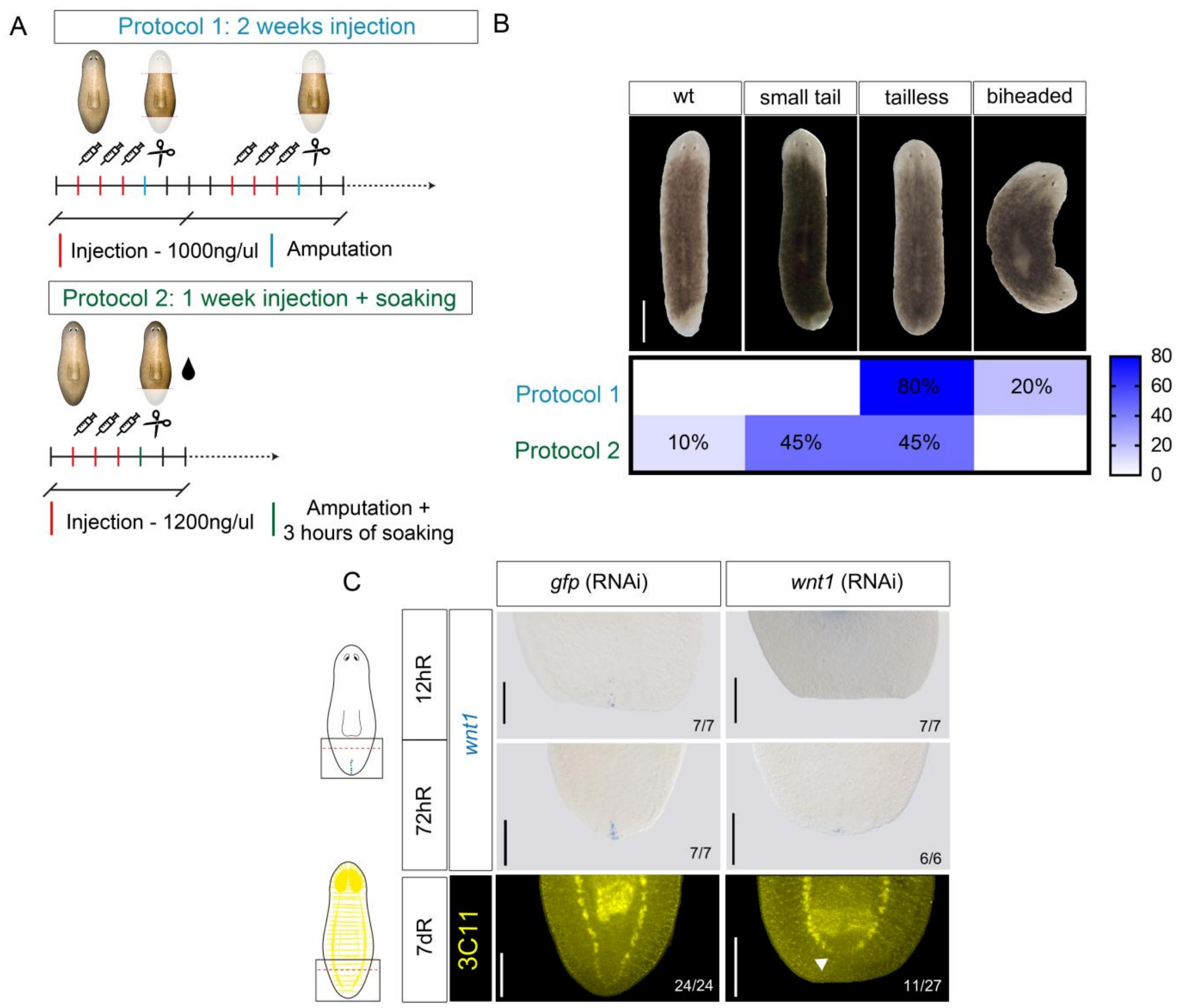

Figure S6. Efficiency of the injection and injection + soaking protocols. (**A**) Schematic representation of the two protocols. (B) Percentage of the phenotypes obtained in each protocol. (**C**) Analysis of the expression of *wnt1* in *wnt1* RNAi animals after the injection + soaking protocol demonstrates that despite a % of animals appear normal, *wnt1* is downregulated in 100% of the animals. Immunostaining with 3C11 demonstrates the tailless phenotype in a % of these animals. Scale bars: 200 µm.

Table Supplementary.

Table Supplementary 1. LRP sequences. In this file we have displayed the genes used to perform the phylogenetic analysis (Figure 1B): the name of the species and its abbreviation, the genes name, its Accession Numbers and the aminoacidic sequences.

Table Supplementary 2. Primers used in this study. We display the name of the primer, their sequences, techniques that has used for and the vector where was cloned.
